# Developing a novel device based on a new technology for non-invasive measurement of blood biomarkers irrespective of skin color

**DOI:** 10.3205/000323

**Published:** 2023-06-23

**Authors:** Sanjay G. Gokhale, Vinoop S. Daggubati, Georgios Alexandrakis

**Affiliations:** 1 Department of Bioengineering, University of Texas at Arlington, Texas, USA; 2 Shani Biotechnologies, LLC, Austin, Texas, USA

**Keywords:** absorption spectrum of hemoglobin, peaks of hemoglobin and oxyhemoglobin, new non-invasive technology and device, remove effect of melanin, one device for all age groups and for all colors, high utility in dark-skinned subjects, wearable technology

## Abstract

**Background:**

Human hemoglobin is a tetrameric metalloporphyrin. The heme part contains iron radicle and porphyrin. The globin part consists of two pairs of amino-acid chains. The absorption spectrum of hemoglobin spans from 250 nm to as high as 2,500 nm, with high coefficients reported in blue and green color zone. The visible absorption spectrum of deoxyhemoglobin has one, while the visible absorption spectrum of oxyhemoglobin shows two peaks.

**Objective:**

(1) To study absorption spectrometry of hemoglobin in 420 to 600 nm range; (2) to conduct preclinical experiments to validate a new device and technology based on green color absorption by hemoglobin; (3) to use this new technology and device for phase 1 study in healthy human volunteers for confirmation

**Design, material and methods:**

(1) Checking absorption spectrometry of hemoglobin in venous blood. We measured absorption spectrometry of 25 mother-baby pairs as an observational study. Readings were plotted from 400 nm to 560 nm. These included peaks, flat lines and deeps. Graph tracings of all samples – cord blood and maternal blood – showed similar patterns. (2) Preclinical experiments were set up (a) to correlate the reflection of green light by hemoglobin and concentration of hemoglobin, (b) to correlate concentration of O_2_ and reflection of green light related to oxyhemoglobin, (c) to correlate concentration of melanin in upper and the hemoglobin in lower layer of tissue phantom and to check the sensitivity of new device with green light for measuring Hb in presence of high levels of melanin, and lastly (d) to check if the new device can measure changes in oxy-hemoglobin and deoxy-hemoglobin, again in presence of high levels of melanin with normal as well as with low levels of hemoglobin. The experiments using bilayer tissue phantom were conducted with horse blood in lower cup as dermal tissue phantom and synthetic melanin in upper layer as epidermal tissue phantom. (3) Phase 1 observational studies following a protocol approved by the institutional review board (IRB) were done in two cohorts. Readings were taken using our device and a commercially available pulse oximeter. In the comparison arm we had Point of Care (POC) Hb test (HemoCu or iSTAT blood test). We had 127 data points of POC Hb test and 170 data points for our device and pulse oximeters. This device uses two wavelengths from the visible spectrum of light and uses reflected light. Light of specific wavelengths is shone on the skin of the individual, and the reflected light is collected as ‘optical signal’. This optical signal – after conversion to electrical signal – is processed and finally analysed with a digital display on the screen. Melanin is accounted using Von Luschan’s chromatic scale (VLS) and a specially designed algorithm.

**Results:**

In this set of various preclinical experiments using different concentrations of hemoglobin and melanin, we indeed demonstrated good sensitivity of our device. It could pick up signals from hemoglobin despite high levels of melanin. Our device is a non-invasive device to measure hemoglobin like a pulse oximeter. Results of our device and pulse oximeter were compared with those by POC Hb test like HemoCu and iSTAT. Our device showed better trending linearity and concordance than a pulse oximeter. Since the absorption spectrum of hemoglobin is the same is new-borns and adults, we could develop one device for all age groups and for people of all colors. Furthermore, the light is shone on the wrist of the individual and is then measured. So, in future this device has the potential of being incorporated in a wearable or smart watch technology.

## Zusammenfassung

**Hintergrund:**

Das menschliche Hämoglobin ist ein tetrameres Metalloporphyrin. Der Häm-Teil enthält Eisen und Porphyrin. Der Globin-Teil besteht aus zwei Paaren von Aminosäureketten. Das Absorptionsspektrum von Hämoglobin reicht von 250 nm bis zu 2.500 nm, mit hohen Koeffizienten in der blauen und grünen Farbzone. Das sichtbare Absorptionsspektrum von Desoxyhämoglobin hat einen Peak, während das sichtbare Absorptionsspektrum von Oxyhämoglobin zwei Peaks aufweist.

**Ziel:**

(1) Untersuchung der Absorptionsspektrometrie von Hämoglobin im Bereich von 420 bis 600 nm; (2) Durchführung präklinischer Experimente zur Validierung eines neuen Geräts und einer neuen Technologie, die auf der Absorption der grünen Farbe durch Hämoglobin basiert; (3) Verwendung dieser neuen Technologie und des Geräts für eine Phase-1-Studie an gesunden menschlichen Probanden zur Bestätigung.

**Studiendesign, Material und Methoden:**

(1) Überprüfung der Absorptionsspektrometrie von Hämoglobin in venösem Blut. Wir haben die Absorptionsspektrometrie von 25 Mutter-Baby-Paaren im Rahmen einer Beobachtungsstudie gemessen. Die Messwerte wurden von 400 nm bis 560 nm aufgezeichnet. Diese beinhalteten Spitzen, flache Linien und Vertiefungen. Die Kurvenverläufe aller Proben – Nabelschnurblut und mütterliches Blut – zeigten ähnliche Muster. (2) Präklinische Experimente wurden durchgeführt, um (a) die Reflexion von grünem Licht durch Hämoglobin und die Hämoglobinkonzentration in Zusammenhang zu setzen, um (b) die O_2_-Konzentration und die Reflexion von grünem Licht im Zusammenhang mit Oxyhämoglobin zu beobachten, um (c) eine Korrelation zwischen der Melaninkonzentration in der oberen und der Hämoglobinkonzentration in der unteren Schicht des Gewebephantoms zu überprüfen sowie die Empfindlichkeit des neuen Geräts mit grünem Licht zur Messung von Hb bei hohem Melaningehalt zu testen und schließlich um (d) zu prüfen, ob das neue Gerät Änderungen von Oxyhämoglobin und Desoxyhämoglobin bei hohem Melaningehalt mit normalem und niedrigem Hämoglobingehalt messen kann. Die Experimente mit einem zweischichtigen Gewebephantom wurden mit Pferdeblut in der unteren Schale als dermales Gewebephantom und synthetischem Melanin in der oberen Schicht als epidermales Gewebephantom durchgeführt. (3) Nach einem von der Ethikkommission genehmigten Protokoll wurden Phase-1-Beobachtungsstudien in zwei Kohorten durchgeführt. Die Messungen wurden mit unserem Gerät und einem handelsüblichen Pulsoximeter durchgeführt. Im Vergleichsarm wurde ein Point-of-Care(POC)-Hb-Test (HemoCu oder iSTAT-Bluttest) durchgeführt. Wir hatten 127 Datenpunkte für den POC-Hb-Test und 170 Datenpunkte für unser Gerät und die Pulsoximeter. Dieses Gerät verwendet zwei Wellenlängen aus dem sichtbaren Spektrum des Lichts und nutzt reflektiertes Licht. Licht bestimmter Wellenlängen wird auf die Haut der Person gestrahlt und das reflektierte Licht wird als „optisches Signal“ erfasst. Dieses optische Signal wird – nach Umwandlung in ein elektrisches Signal – verarbeitet und schließlich mit einer digitalen Anzeige auf dem Bildschirm analysiert. Das Melanin wird mithilfe der Von-Luschan-Skala (VLS) und eines speziell entwickelten Algorithmus erfasst.

**Ergebnisse:**

In dieser Reihe von verschiedenen präklinischen Experimenten mit unterschiedlichen Konzentrationen von Hämoglobin und Melanin haben wir eine gute Empfindlichkeit unseres Geräts nachgewiesen. Es konnte Signale von Hämoglobin trotz hoher Melaninkonzentrationen aufnehmen. Unser Gerät ist wie ein Pulsoximeter ein nicht-invasives Gerät zur Messung von Hämoglobin. Die Ergebnisse unseres Geräts und des Pulsoximeters wurden mit denen von POC-Hb-Tests wie HemoCu und iSTAT verglichen. Unser Gerät zeigte eine bessere Trendlinearität und Konkordanz als ein Pulsoximeter. Da das Absorptionsspektrum von Hämoglobin bei Neugeborenen und Erwachsenen das gleiche ist, konnten wir ein Gerät für alle Altersgruppen und für Menschen aller Hautfarben entwickeln. Außerdem wird das Licht auf das Handgelenk der Person gestrahlt und dann gemessen. In Zukunft könnte dieses Gerät also in eine tragbare oder intelligente Uhrentechnologie integriert werden.

## Introduction

Hemoglobin monitoring is a common laboratory test in all healthcare facility, including hospitals and ICUs, and is indicated in many clinical conditions. Needle prick or a blood draw is the usual method of choice. Another option is non-invasive pulse oximetry. All these have their own merits and demerits. Blood draw and needle pricks, though accurate, are painful and carry the risk of infections. This demands a phlebotomist and clinical laboratory facility with trained staff, which adds to the cost of healthcare. A pulse oximeter using two wavelengths in red and infrared spectrum works on the principles of photoplethysmography and ‘transmittance of light’. The oximeter is suitable for routine examinations, but its use is questionable in an emergency [1], [2]. These inaccuracies are more marked in people of color, and even the US senate took a note of it [3].

In the visible range, the main chromophores of human skin are hemoglobin and melanin. Hemoglobin is found in the microvascular network of the dermis, typically 50±500 µm [up to 0.5 mm] below the skin surface. In contrast, melanin is in the epidermis, which occupies the top 50±100 µm [up to 0.1 mm]. Melanin competes with hemoglobin for absorption of electromagnetic radiations from the visible spectrum of 450 to 580 nm [4]. Hemoglobin absorbs blue, green, and yellow light well and red light poorly [5].

With this background, a new non-invasive device is developed to measure blood biomarkers by using two diodes of two specific wavelengths from 450 to 580 nm, to measure hemoglobin noninvasively, accounting for melanin in skin and overcoming the racial bias in measurements.

This device works on the principles of reflectance of light. Light of specific wavelengths is shone on the skin of the individual and the reflected light is collected as ‘optical signal’. This optical signal – after conversion to electrical signal – is processed and finally analysed with a digital display on the screen. Melanin is accounted using Von Luschan’s chromatic scale (VLS) and a specially designed algorithm. In the background we did absorption spectrometry of 25 mother-baby pairs as an observational study. Interestingly, graph tracings of all samples – cord blood and maternal blood – showed similar patterns [6].

**Figure 1:**
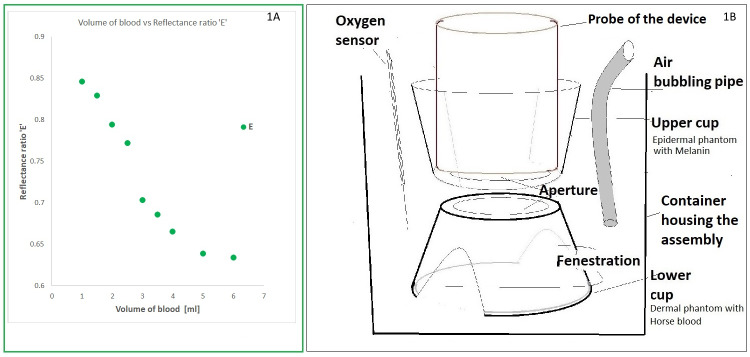
Validation of device for hemoglobin measurements. A: Volume of blood or hemoglobin vs. reflectance ratio ‘E’. B: Preclinical experimental setup

Preclinical experiments using ‘two cup assembly and bilayer tissue phantom’ were conducted with horse blood in lower cup as dermal tissue phantom and synthetic melanin in upper layer as epidermal tissue phantom (Figure 1 (Fig. 1)). The results of the preclinical or tissue phantom studies using bilayer tissue phantom were all positive, and later this novel device was tested in two cohorts at the University of Texas at Arlington. Candidates with different skin color and from different ethnicities were enrolled. Readings were taken using our device and a commercially available pulse oximeter. Our device is a non-invasive device to measure hemoglobin like a pulse oximeter. HemoCu and iSTAT are both blood draw methods. Results of our device and pulse oximeter were compared with those by Point of Care (POC) Hb test. Our device with our new technology (Patent granted by USPTO) showed better trending linearity and concordance than the pulse oximeter. Our device appeared more accurate than the pulse oximeter. Secondly, the light shone on the wrist of the individual is measured. So, in future this device has the potential to be incorporated in a wearable or smart watch technology.

## Material and methods

The studies were done in three parts.

**Part 1: **Checking absorption spectrometry of hemoglobin in venous blood

**Part 2:** Preclinical or tissue phantom studies

**Part 3:** Clinical studies: Later in the third part, the device was tested in healthy human volunteers in two cohorts. Melanin was accounted for using VLS scale or chart.

### Part 1: Checking absorption spectrometry of hemoglobin in venous blood

There are scant reports in which the molecular structure of Hb-A and Hb-F was studied using absorption spectroscopy. We have studied the absorption spectrometry of 25 mother-baby pairs in detail. Readings were plotted from 400 nm to 600 nm. These included peaks, flat lines and deeps. Graph tracings of all samples – cord blood and maternal blood – showed similar patterns. Interestingly, despite different globin chains, cord hemoglobin almost copied the performance of maternal hemoglobin. The graphs appeared nearly identical and all samples, whether maternal or neonatal, showed nearly identical peaks [6]. 

### Part 2: Preclinical or tissue phantom studies

Preclinical studies using bilayer tissue phantom were conducted with horse blood in lower cup as dermal tissue phantom and synthetic melanin in upper layer as epidermal tissue phantom. These were done (1) to correlate the reflection of green light by hemoglobin and concentration of hemoglobin, (2) to correlate concentration of O_2_ and reflection of green light related to oxyhemoglobin, (3) to correlate concentration of melanin in upper and the hemoglobin in lower layer of tissue phantom and to check the sensitivity of new device with green light for measuring Hb in presence of high levels of melanin, and lastly (4) to check if the new device can measure changes in oxy-hemoglobin and deoxy-hemoglobin, again in presence of high levels of melanin with normal as well as with low levels of hemoglobin. In this set of various experiments using different concentrations of hemoglobin and melanin, we indeed demonstrated good sensitivity of our device. It could pick up signals from hemoglobin despite high levels of melanin. This is indeed an innovative approach for non-invasive measurements of hemoglobin and oxyhemoglobin using this new concept of bilayer tissue phantom. After these experiments in preclinical study, we could redesign and modify our probe with certain modifications for phase 1 study in two cohorts. And indeed, we ended with promising results in both cohorts (patent granted by United States Patent and Trademark Office).

These experiments were done at the University of Texas at Arlington (Department of Bioengineering) from November 2020 to April 2021.

1. Materials
2. Making a tissue phantom
3. Setting up the device
4. Experimental setup
5. Measurements

#### 1 Materials

The following materials were used in these experiments: defibrinated sterile horse blood (HemoStat Laboratories), 20% Intralipid, DW or distilled water, yeast, synthetic melanin (SCBT) by Sigma Aldrich in 1% H_2_O_2_, diluted further as needed. Additionally, plastic cups to make 2 cup assembly, rectangular plastic containers, and a polyethylene sheet 5 microns thick were used in different combinations in these experiments. 

Following equipments and instruments were also used: ‘PreSens’ oxygen measurement sensor (PreSens Precision Sensing GmbH, Am BioPark 11, 93053 Regensburg, Germany), air bubbling pipes, and our new device.

#### 2 Making a tissue phantom

We have mimicked blood in dermis of skin, using defibrinated sterile horse blood (HemoStat Laboratories), 20% Intralipid in suitable dilutions as scattering agent, DW or distilled water as diluent. Blood was used in 1.5%, 2% or 2.5% volume concentrations mimicking blood in dermis. 

**Preparing dermal and epidermal tissue phantom: **Dermal tissue phantom – 20% Intralipid – was diluted with DW to make 400 ml of dermal tissue phantom in a white plastic rectangular container with a height of about 12 to 15 cm. The rectangular container was preferred so as to position air bubbling pipe and oxygen probe at 180 degrees to each other (next experiments). The rectangular shape helped to secure/fix the air bubbling pipe and oxygen probe using adhesive tapes. This tissue phantom mimics human tissues in terms of scattering. This cup mimicking dermis was in lower position. The upper cup was the epidermal tissue phantom. 

Epidermal tissue phantom-Intralipid was used in same formula for epidermal tissue phantom.

**Making hydrogen peroxide 1% solution: **3% H_2_O_2_ was diluted with DW (13 ml of 3% H_2_O_2_+27 ml DW to make 40 ml of 1% H_2_O_2_ solution). 

**Preparing cup for epidermal tissue phantom:** Disposable transparent plastic cups were used for this purpose. Diameter at bottom of cup measured 2.5 inches. Central circular part about 1.25 to 1.5 inch diameter was excised. The cup was then capped with thin transparent plastic/a polyethylene pouch. The pouch was held in place with rubber bands and adhesive water-resistant tape. 

**Preparing melanin epidermal phantom:** Synthetic melanin [SCBT] was used in 1% H_2_O_2_. Different concentrations of melanin (mg/ml melanin wt/volume solution) were used to make test solutions (Table 1 (Tab. 1)).

**Preparing stand/stage** for placing green light probe in position. As shown in Figure 1B (Fig. 1), a stand was made using a plastic cup. First cup is in inverted position/upside down. This is the base/stand/support for upper cup holding epidermal melanin tissue phantom. A circular hole is drilled in the base. The diameter is about 2 inches. The perimeter wall is fenestrated on two sides, 180 degrees apart. This facilitates free flow of fluid/liquid. The cup is cut at the height of 2 cm. 

Upper cup is placed on it in upright position. This is drilled at the base with a circle of the same size. It has a height of about 5.5 inches. Lower cup is placed in the container holding Intralipid and water solution (dermal tissue phantom). Upper cup with epidermal melanin tissue phantom is mounted on first one in upright position as described (Figure 1B (Fig. 1)). The probe of our device is then placed in upper cup.

**Preparing green light probe/our device:** To prevent accidental damage by contact with solution/water to light emitting diodes, the light emitting diode end of our device/bottom part was well covered with a transparent thin plastic/polyethene pouch. The pouch was kept in place with a rubber band. Thickness of plastic pouch was 5 divisions by micrometre, 5 microns. 

#### 3 Setting up the device

The device LEDs were put on and the sensor circuit was activated. A polythene cover 5 microns thick was put on the front part of the device to prevent damage to internal circuits by leakage of solutions/liquids in the probe. Initial baseline readings were taken against standard surface for 525 nm and 555 nm wavelengths; this serves as the calibration reading.

#### 4 Experimental setup

**Figure 2:**
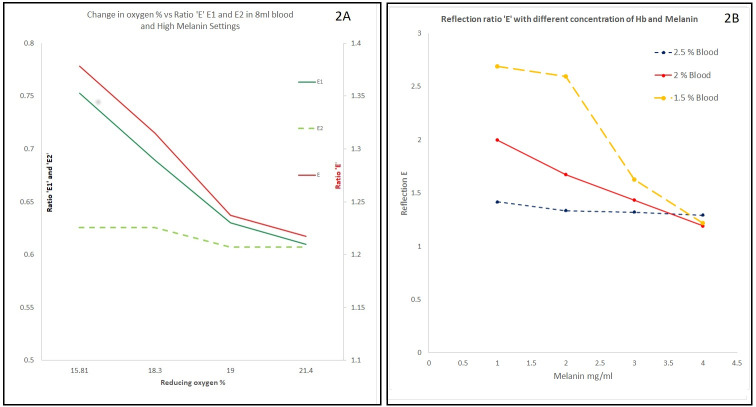
A: Preclinical experiment ‘Reducing oxygen %’ vs. reflectance ratio ‘E1’, ‘E2’ and ‘E’. B: Change in reflectance ratio with change in concentration of hemoglobin and/or melanin

Different experiments were designed using our device (Figure 1 (Fig. 1) and Figure 2 (Fig. 2)).

Exp-1: To measure the effect of increasing Hb concentration on the reflected light 

Exp-2: To study effects of bubbling air in horse blood samples on ratio E, E1, and E2 

Exp-3: To measure the effect melanin concentration on the device measured ratio E 

Exp-4: To study effects of O_2_% on ratio E, E1, and E2 and change in concentrations of oxy-hemoglobin and deoxy-hemoglobin in bilayered tissue phantom of horse blood and synthetic melanin 

#### 5 Measurements

In all the experiments baseline reflections were noted on reference or standard surface for both LEDs. Ratio of reflection from first LED 525 nm divided by reflection from reference surface was labelled as E1 and that from second LED 555 nm as E2. E1 and E2 added together to give total ratio or E. The probe was held in a fixed position and reflections were noted at all the instances.

### Part 3: Clinical studies

Later in the third part the device was tested in healthy human volunteers. The confounding effect of melanin was considered and as for measurement of melanin, we used the VLS. The color of skin on the back of the wrist was compared with the color images of VLS.

This novel device was tested in two cohorts, and two studies approved by the Institutional Review Board (IRB) were done at the University of Texas at Arlington. Readings were taken using our device and a commercially available pulse oximeter. In the comparison arm we had POC Hb test (HemoCu or iSTAT blood test). We had 127 data points of POC Hb test and 170 data points for our device and pulse oximeters. Light of specific wavelengths is shone on the back of the wrist of the subject and the reflected light is collected as ‘optical signal’. This optical signal – after conversion to electrical signal – is processed and finally analysed with a digital display on the screen. Melanin is accounted for using VLS and a specially designed algorithm. This device uses two wavelengths from the visible spectrum of light and uses reflected light [7]. Our device is a non-invasive device to measure hemoglobin like a pulse oximeter. HemoCu and iSTAT are both blood draw methods. Results of our device and pulse oximeter were compared with those by POC Hb test. Our device showed better trending linearity and concordance than the pulse oximeter. Our device appeared more accurate than the pulse oximeter. 

## Discussion

Normal adult hemoglobin (Hb A) is a protein consisting of four polypeptide chains, each of which is bound to a heme. The heme in Hb A is named iron-protoporphyrin and is responsible for most of the light absorption in blood. Hemoglobin (Hb) is a well-known O_2_-transporting protein. Human adult Hb (Hb A) is a heterotetramer consisting of two ‘α’ alpha and two ‘β’ beta globin chains, and hemoglobin-F or fetal hemoglobin has two ‘α’ alpha and ‘γ’ gamma globin chains. Deoxyhemoglobin binds to oxygen and becomes oxyhemoglobin. The de-oxy and oxygenated states show some differences at molecular level. The energy level increases during transition to oxyhemoglobin [8], [9]. Shortly after birth there is a switch from HbF to adult hemoglobin (HbA). HbF is highest (98%) at birth, decreasing at 5% per week till 6 months when it wanes off so that by 6 months of age the major hemoglobin is HbA.

As said earlier, the absorption spectrum of hemoglobin spans from 250 nm to as high as 2500 nm, but high coefficients or high peaks are reported in blue and green color zone [8]. The visible absorption spectrum of deoxyhemoglobin has one, while the visible absorption spectrum of oxyhemoglobin shows two peaks [9]. In our study of absorption spectrometry of 25 mother-baby pairs, all blood samples showed Soret band of same peak and wavelength. This is the porphyrin part, common to all hemoglobins [10], [11]. Fully oxygenated hemoglobin shows beta band at 540 nm and alpha band at 576 nm [12], but interestingly, both neonatal and maternal, being venous blood samples, had identical peaks. As discussed earlier, the energy level of hemoglobin is high in oxygenated state vs. deoxygenated state. Cord blood is venous or deoxygenated and maternal sample was obviously venous one. Blood in the de-oxygenated state fails to show the different peaks characteristic of various globin chains. Therefore, we got the same graph for cord and maternal blood samples [6].

In the preclinical tissue phantom experiments we measured reflection of green light. Total absorbance correlates with the total hemoglobin concentration. Reflectance bears an inverse relation [13], [14]. Increased blood in dermal phantom or increased melanin concentration in epidermal tissue phantom causes a drop in reflectance [15]. As said earlier, hemoglobin absorbs green light well and red light poorly. Melanin absorbs green, red, and infrared light [5]. Hemoglobin has a strong Soret band absorption peak at 420 nm in blue light zone, which overlaps with the bilirubin absorption peak at 460 nm [16]. Absorption spectra of oxyhemoglobin shows different peaks than deoxyhemoglobin. Absorbance spectroscopy, based on the Beer-Lambert law, is effective for measuring the quantity of a particular chemical in a given medium [17], [18], [19]. The greatest region of dissimilarity between oxy and deoxyhemoglobin is from 520 to 580 nm [9], [15]. Interestingly, green light goes only 2.5 to 3 mm deep in contrast to red light, which penetrates up to 5 mm. The penetration of green light is 60% of that of red light, but the absorption of green light is 20 times more than that of red light, and green light can detect superficial tissues better [20], [21]. Melanin and hemoglobin are important chromophores for the absorption of green light. We did an absorption scan of synthetic melanin and plotted concentrations of melanin mg/ml vs. coefficient of absorption (Table 1 (Tab. 1)). In dark-skinned subjects, coefficient of absorption of melanin at 694 nm is 2.5/cm [22]. The corresponding figure for 555 nm is 3.2/cm, but in these experiments, we reached coefficient of absorption of melanin at 555 nm to almost 4.01. This is way ahead of reference values for dark-skinned individuals (Table 1 (Tab. 1)).

While using green light, we focused only on the absorption of light. The primary optical function of melanin is absorption, and Rayleigh scattering in melanin is negligible [23].

In the preclinical tissue phantom experiments our probe could measure reflection of green light from dermal or lower cup holding blood in the presence of high levels of melanin in epidermal or upper cup. In other experiments with increasing levels of O_2_% more oxyhemoglobin was produced with more absorption and lesser reflection of green light. So, the reflectance or ratio E1 and E decreased with increasing levels of O_2_%. This was like pulmonary circulation or loading of hemoglobin with oxygen. Later, when yeast was added, oxygen sensor recorded decreasing levels of oxygen in dermal phantom and increase in reflectance of green light with higher ratio E1 and E as measured on multimeter as higher electrical signal. Here, we simulated the scenario of peripheral circulation at tissue level. We could easily recreate the scenario of loading and unloading of oxygen or pulmonary and peripheral circulation in health or under physiological conditions and in conditions mimicking anaemia in dark-skinned subjects.

**Figure 3:**
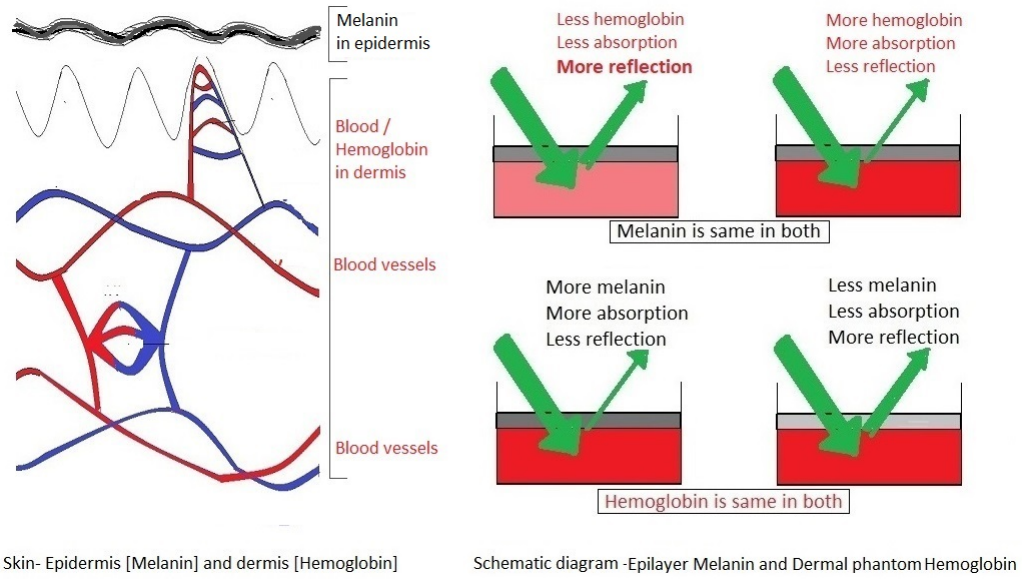
Reflection of light is inversely proportional to the amount of hemoglobin in dermis and/or melanin in epidermis

After the preclinical studies this new technology and the novel device were tested with certain modifications in two cohorts in studies approved by the IRB at the University of Texas at Arlington. This new device for non-invasive estimation of hemoglobin is developed using two specific wavelengths from 450 to 580 nm, measuring reflected light. Light of specific wavelengths is shone on the skin of the individual, and the reflected light is collected as ‘optical signal’ [7]. Total absorbance correlates with the total hemoglobin concentration. Reflectance bears an inverse relation (Figure 3 (Fig. 3)) [13], [14]. Increased hemoglobin in dermal tissue or increased melanin concentration in epidermal tissue caused a drop in reflectance (Figure 2B (Fig. 2) and Figure 3 (Fig. 3)) [15]. This optical signal – after conversion to electrical signal – is processed and finally analysed with a digital display on the screen. Melanin is accounted using Von Luschan Chromatic Scale (VLS) and a specially designed algorithm. This device uses two wavelengths from visible spectrum of light and uses reflected light. Readings were taken using our device and a commercially available pulse oximeter. In the comparison arm we had POC Hb test (HemoCu or iSTAT blood test). We had 127 data points of POC Hb test and 170 data points for our device and pulse oximeters.

To account for melanin, we used VLS. Prior to the introduction of reflectance spectrophotometry into anthropological field research during the 1950s, human skin color was most commonly classified by visual skin color matching using the Von Luschan tiles, a set of 36 standardized, opaque glass tiles arranged in a chromatic scale [24]. The validated VLS is used to assess the skin color or skin tone melanin content. VLS is an easy to use, clinical measure of the relative melanin content (color tone) in the skin. It consists of numeric values from 1 to 36, with higher values representing more melanin content (darker tone) in the skin.

## Conclusion

Our device is a non-invasive device to measure hemoglobin similar to a pulse oximeter. HemoCu and iSTAT are both blood draw methods. Results of our device and pulse oximeter were compared with those of POC Hb test. 

**Figure 4:**
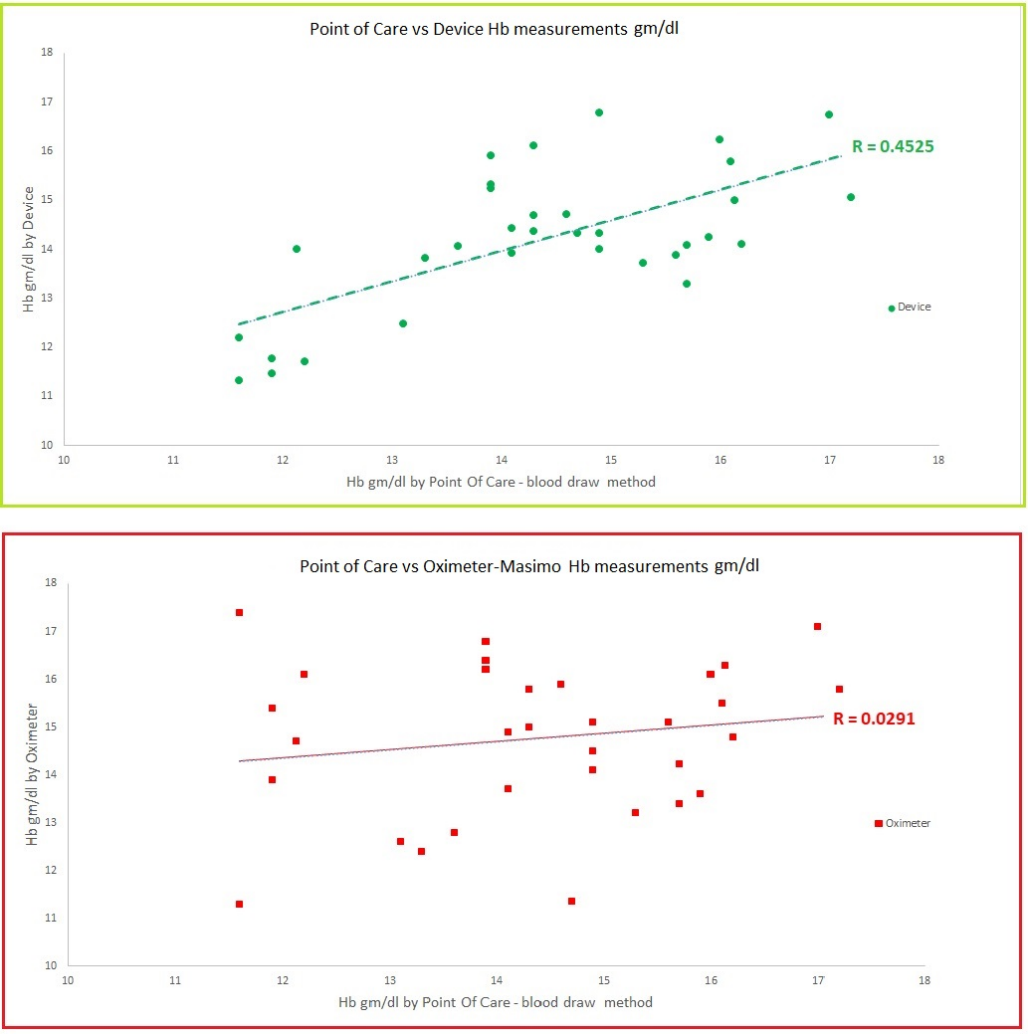
Comparing Hb gm/dl by Point of Care (POC) blood draw vs. the new device and oximeter

Our device showed better trending linearity and concordance than the pulse oximeter (Figure 4 (Fig. 4)). Our device appeared more accurate than the pulse oximeter. Strengths of the study include a sizable number of observations (about 170) in subjects with diverse skin melanin content. Data integrity was ensured by real time electronic recording with timestamps. The study had a relatively small number of healthy subjects. Larger studies involving patients with variable degree of hypoxia are needed for further development and validation of this device. Our preliminary findings support a potential new technology for the assessment of capillary oxygen. The technology and the device are non-invasive and could potentially overcome current limitations of the pulse-oximetry devices.

This novel device uses simple optics and targets superficial part of skin and capillaries. Our technology offers an additional advantage to overcome the impact of skin color (melanin) during the estimation process. We have developed our algorithm and mathematical formulae to account for melanin effects. This is very simple to use. The device has a very specific geometric design. One device can be used in all age groups from newborns to nonagenarians. Since it is battery operated, it can be used in field work. The data is stored and can be transferred easily. Secondly, the light is shone on the wrist of the individual, which is then measured. So, in future this device has the potential of being incorporated in a wearable or smart watch technology.

**Highlights**

1. The study confirms the absorption spectrometry of hemoglobin in a range of 420 to 600 nm.
2. Preclinical studies were conducted to validate a novel device based on a new technology for non-invasive measurement of hemoglobin. These tissue phantom experiments using bilayer tissue phantom and two cup assembly were conducted with horse blood in lower cup as dermal tissue phantom and synthetic melanin in upper layer as epidermal tissue phantom.
3. In this set of various experiments using different concentrations of hemoglobin and melanin, we demonstrated good sensitivity of our device. It could pick up signals from hemoglobin in dermal phantom despite high levels of melanin in the epilayer.
4. We could mimic loading and unloading of O_2_ as seen in pulmonary and peripheral circulation. This is indeed an innovative approach for non-invasive measurements of hemoglobin and oxyhemoglobin using this new concept of bilayer tissue phantom.
5. After these experiments in the preclinical study, we redesigned and modified the probe of our device for phase 1 study in two cohorts. And indeed, we achieved promising results in both cohorts, and further clinical studies are on the way.

**New findings**

1. This is the first ever use of green light (520–570 nm) in a non-invasive hemoglobin and oximetry device.
2. The new device eliminates the impact of skin color on the observations.
3. It can be potentially used in adults, children, and neonates: one device for all.
4. It can be used in wearables and low resource settings.

**How it might impact future healthcare**

1. The new device provides a reliable, non-invasive tool for hemoglobin and oxygen saturation monitoring in several settings (home, outpatient clinics, emergency room and intensive care units, and wearables).

## Notes

### Patent

Patent granted: US11191460B1

### Ethical approval

The study was approved by the Institutional Review Board (IRB) at the University of Texas at Arlington: STU-2021-0150, approval date: 02/23/2021.

A written, informed consent was obtained from each participant. Copies of the consent forms are with IRB authorities.

### Funding

The project was funded by Shani Biotechnologies, LLC, Austin, TX 78701, USA.

Dr. Georgios Alexandrakis received funding from Shani Biotechnologies, LLC, Austin, TX 78701, USA. Dr. Sanjay G. Gokhale did not receive any funding.

### Authors’ contributions

Dr. Sanjay G. Gokhale conceptualized and designed the study, drafted the initial manuscript, monitored the site, and approved the final manuscript as submitted.

Dr. Vinoop S. Daggubati drafted the initial manuscript and approved the final manuscript as submitted.

Dr. Georgios Alexandrakis drafted the initial manuscript and approved the final manuscript as submitted.

### Competing interests

The authors declare that they have no competing interests.

## Figures and Tables

**Table 1:**
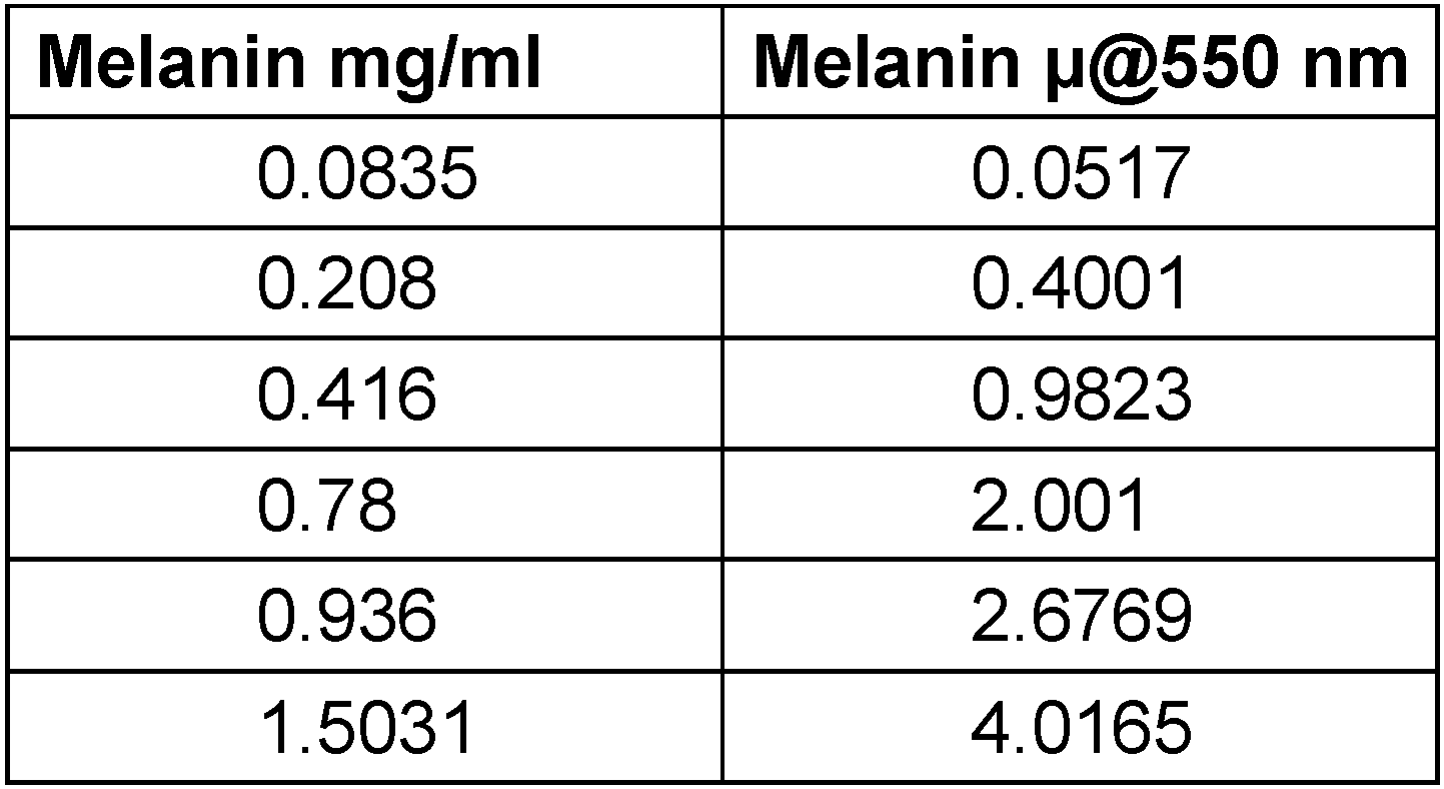
Melanin mg/ml vs absorption coefficient of melanin µ @ 550 nm
